# Improving intraoperative diagnosis of spread through air spaces: A cryo-embedding-medium inflation method for frozen section analysis

**DOI:** 10.1016/j.xjtc.2024.02.014

**Published:** 2024-03-01

**Authors:** Takashi Eguchi, Shunichiro Matsuoka, Mai Iwaya, Shota Kobayashi, Maho Seshimoto, Shuji Mishima, Daisuke Hara, Hirotaka Kumeda, Kentaro Miura, Kazutoshi Hamanaka, Takeshi Uehara, Kimihiro Shimizu

**Affiliations:** a Division of General Thoracic Surgery, Department of Surgery, Shinshu University Hospital, Matsumoto, Japan; b Department of Laboratory Medicine, Shinshu University Hospital, Matsumoto, Japan

**Keywords:** lung cancer, intraoperative decision-making, spread through air spaces lesions, cryo-embedding

## Abstract

**Objective:**

Accurate intraoperative diagnosis of spread through air spaces (STAS), a known poor prognostic factor in lung cancer, is crucial for guiding surgical decision-making during sublobar resections. This study aimed to evaluate the diagnostic sensitivity of STAS using frozen section (FS) slides prepared with the cryo-embedding medium inflation technique.

**Methods:**

In this prospective study at Shinshu University Hospital, 99 patients undergoing lung resection for tumors <3 cm in size were included, a total of 114 lesions. FS slides were prepared with injecting diluted cryo-embedding medium into the lung parenchyma of resected specimens. The diagnostic performance of these FS slides for STAS detection was evaluated by comparing FS-STAS results with the gold-standard STAS status.

**Results:**

The incidence of STAS, determined by the gold standard, was 43 (38%) of 114 lesions, including 31 (37%) of 84 primary lung cancers and 12 (40%) of 30 metastatic lung tumors. The sensitivity, specificity, positive and negative predictive values, and accuracy of FS slides for STAS detection were 81%, 89%, 81%, 89%, and 86%, respectively. Specifically, in primary lung cancers, these values were 90%, 89%, 82%, 94%, and 89%, respectively. Regarding metastatic lung tumors, the corresponding values were 58%, 89%, 78%, 76%, and 77%, respectively.

**Conclusions:**

Our adapted cryo-embedding medium inflation method has demonstrated enhanced sensitivity in detecting STAS on FS slides, providing results similar to the gold-standard STAS detection. Compared with historical benchmarks, this technique could show excellent performance and be readily incorporated into clinical practice without requiring additional resources beyond those used for standard FS analysis.

## Graphical abstract

**Figure:**
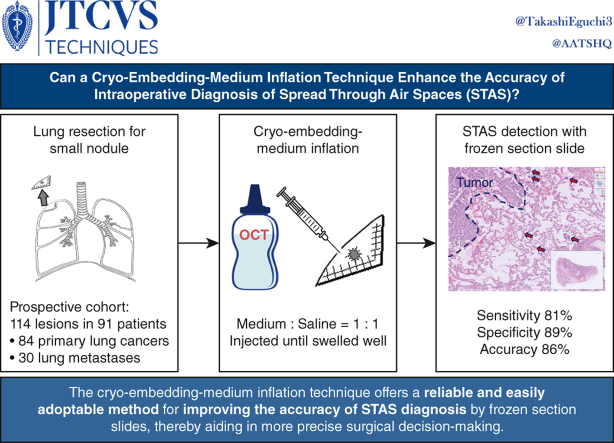

**Figure:**
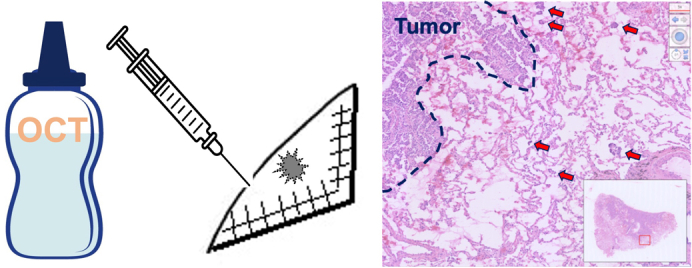
Frozen section slide preparation process to detect tumor spread through air spaces (STAS).

**Central Message**

A cryo-embedding-medium inflation technique could improve intraoperative diagnosis of spread through air spaces of tumors on frozen section slides. It could be both effective and easily implementable.

**Perspective**

We adapted and refined the established cryo-embedding-medium inflation method to address the limitations of the current frozen section analysis for detecting spread through air spaces. This study may support critical intraoperative decisions with direct implementation using common histopathologic resources.

Detection of early-stage lung cancer and the opportunity for surgical intervention in older patients are increasing. Traditionally, lobectomy is the standard surgical treatment of early-stage lung cancer.^1^ Recently, 2 large randomized trials have compared the primary oncological and functional outcomes of sublobar lung resection, including segmentectomy and wedge resection, for early-stage lung cancer with those of lobectomy. These trials revealed that sublobar resection was associated with superior or noninferior long-term survival compared with lobectomy (JCOG0802/WJOG4607L^2^ and CALGB/Alliance 140503^3^). Consequently, sublobar resection for early-stage lung cancer is becoming the standard curative-intent surgical treatment, and its use is expected to increase.

Spread through air spaces (STAS) is a well-established poor prognostic factor, particularly in patients undergoing sublobar resection.^4^, ^5^, ^6^ Accurate preoperative or intraoperative diagnosis of STAS could inform surgical decision-making, such as choosing between lobectomy and sublobar resection. However, previous attempts to diagnose STAS intraoperatively using frozen section (FS) analysis were unsuccessful because of its relatively low sensitivity (44%-55%).^7^^,^^8^ In contrast, another study reported better sensitivity (71%) in detecting STAS using FS slides based on analyzing tumor slides with adequate and well-expanded lung parenchyma surrounding tumors.^5^

We hypothesized that the limited sensitivity of FS slides in detecting STAS might be caused by collapse of the parenchyma adjacent to tumor. To improve parenchymal expansion, we adapted the established cryo-embedding medium inflation techniques.^9^^,^^10^ Initially introduced by Gianoulis and colleagues^9^ in 1988 to minimize specimen shattering on FS of the lung, and later refined to differentiate lepidic from invasive adenocarcinoma lesions,^10^ these techniques have not yet been evaluated for STAS assessment using FS slides. Our study aimed to bridge this gap by investigating the utility of these techniques in STAS detection.

## Methods

### Study Cohort

This single-center prospective study was approved by the institutional review board of Shinshu University Hospital (Project ID, 5013; approved on January 27, 2021). All included patients provided written informed consent for publication of study data. Between February 1, 2021, and December 31, 2021, 210 patients underwent lung resection for lung nodules smaller than 3 cm at Shinshu University Hospital.

**Figure 1:**
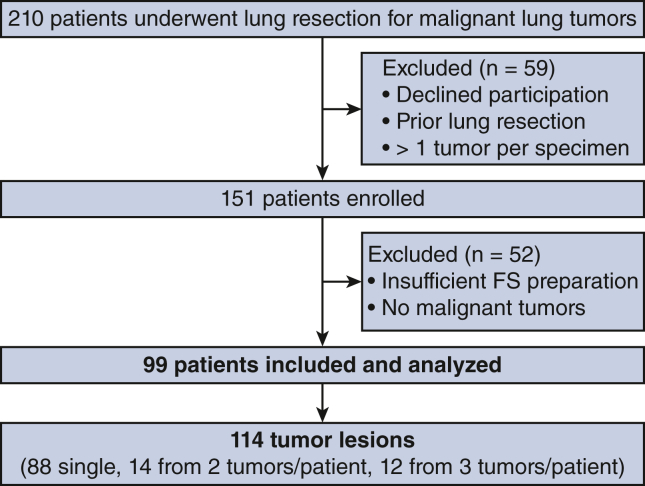
Patient selection flowchart. *FS*, Frozen section.

The study included any type of lung resection, such as wedge resection, segmentectomy, or lobectomy. Patients were excluded if they did not provide informed consent, had a history of lung resection, or exhibited more than 1 tumor in a single resected specimen. However, patients undergoing individual resections of multiple tumors were included. Patients requiring intraoperative tumor diagnosis based on FS analysis were excluded from this study. This exclusion was the result of our study design, which did not incorporate real-time FS analysis on the day of surgery, as described in the section to follow. Figure 1 illustrates the study flowchart.

### FS Slide Preparation Using the Cryo-Embedding-Medium Inflation Technique

Tumor lesions were excised using minimally invasive surgery, specifically, wedge resection, segmentectomy, or lobectomy. During surgery, the lung parenchyma was sectioned using either a surgical stapler or an electrocautery. The surgical specimens were immediately transported from the operating room to the pathology department in sterile bags.

Upon receipt of the resected specimens, pathology technicians and pathologists proceeded with the preparation of FS slides. The process involved injecting a 1:1 dilution of cryo-embedding medium (Tissue-Tek O.C.T. Compound; Sakura Finetek) and saline into the lung parenchyma of the resected specimen until ensuring sufficient lung swelling. “Sufficient lung swelling” was defined as an expansion level similar to the lung during inhalation. This criterion was adhered to ensure a consistent and reproducible technique for preparing slides. To optimize tissue handling and diagnostic clarity, we conducted preliminary experiments to determine the ideal solution ratio for the cryo-embedding medium. We considered factors such as tissue integrity, medium penetration, and ease of preparation. Our findings indicated that a 1:1 solution ratio was effective in highlighting STAS in the lung parenchyma.

**Figure 2:**
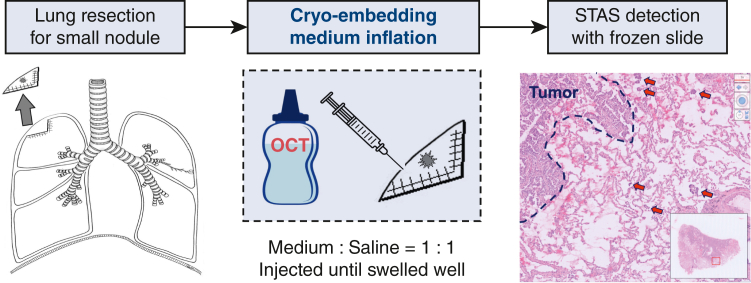
A schematic representation of the frozen section slide-preparation process. *STAS*, Spread through air spaces.

Subsequently, a frozen tissue block comprising tumor area and the surrounding parenchyma was obtained. Subsequently, 5-μm thick FS slides were obtained using a cryostat and then stained with hematoxylin–eosin, in accordance with standard procedures. Figure 2 shows a schematic of the FS slide-preparation process.

### Definition of STAS

STAS was defined as presence of tumor cells within air spaces located beyond the edge of the main tumor, manifesting as clusters, solid nests, or aggregates of single cells. This definition adheres to the criteria established by Kadota and colleagues.^4^ In our study, we exercised caution by not considering tumor cells within the alveolar spaces immediately adjacent to the estimated border of tumor as STAS. This was to avoid potential misinterpretation of cells at the tumor edge as STAS, thereby ensuring a more precise and accurate identification.

### Histologic Evaluation of STAS Using FS Slides

**Figure 3:**
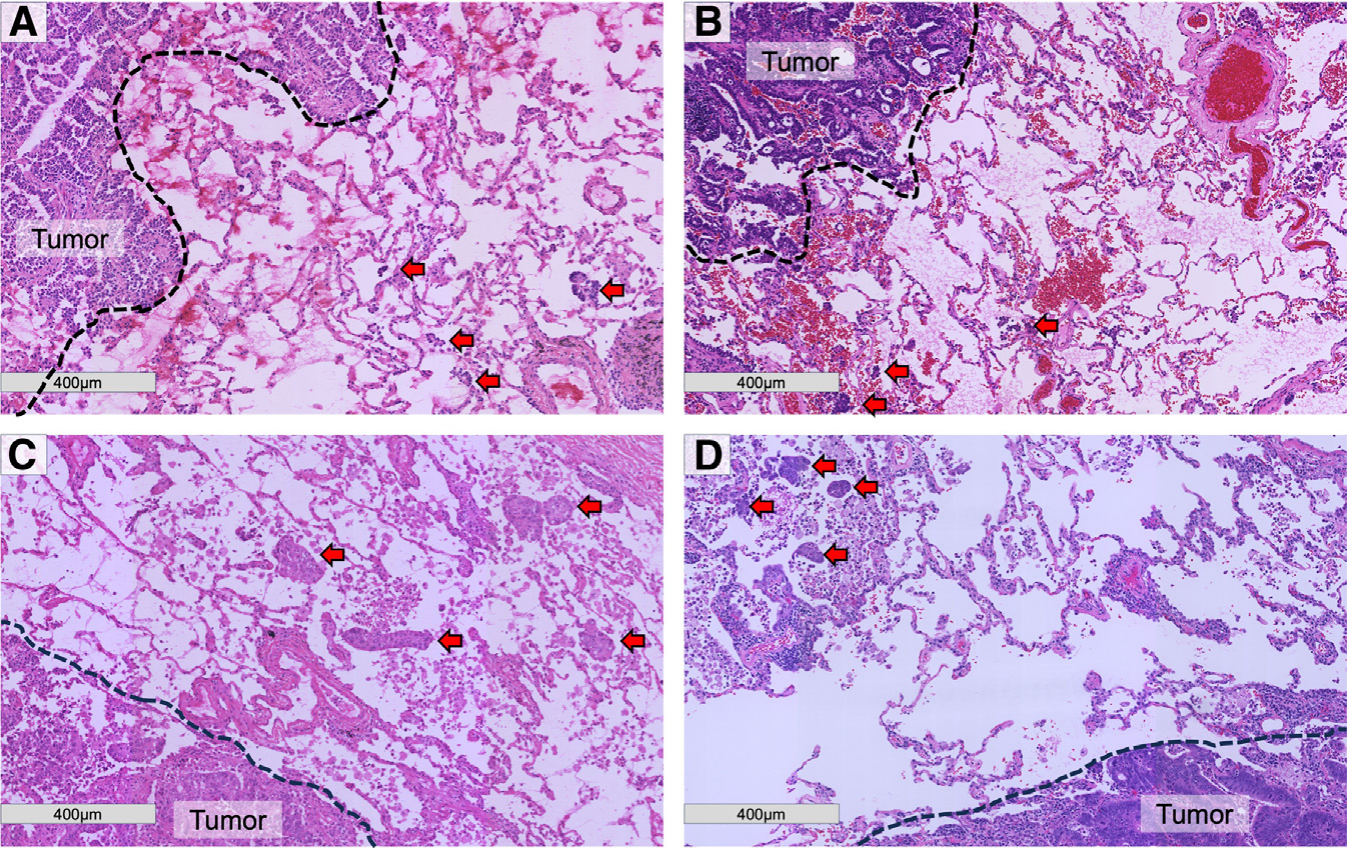
Hematoxylin–eosin staining images of frozen section and permanent slides from representative cases. A, A frozen section slide from a patient with primary lung adenocarcinoma. B, A permanent slide from a patient with primary lung adenocarcinoma C, A frozen section slide from a patient with metastatic lung tumor (colon primary) D, A permanent slide from a patient with metastatic lung tumor (colon primary). *Dotted lines* and *gray arrows* represent tumor edge and STAS areas, respectively. *STAS*, Spread through air spaces.

The histologic evaluation to determine the STAS status using FS slides was performed independently of the surgical procedure on a separate day. This approach was chosen as the study was designed to evaluate the efficacy of the cryo-embedding-medium inflation technique in STAS diagnosis, independent of immediate clinical decision-making. However, the preparation of the FS slides proceeded immediately upon receipt of the resected specimens on the day of surgery, as previously described. To maintain objectivity in the assessment of STAS on FS slides, the pathologists were blinded to patient medical information, including the results of permanent slides. Moreover, the medical staff members who were either directly or indirectly involved in patient care were not involved in the histologic evaluation. Three pathologists (M.I., T.U., and S.K.) individually assessed FS slides to determine the presence or absence of STAS (FS-STAS status). Highlighting that consensus meetings were only conducted during the preliminary phase of the study, not during the analysis of the 114 sessions included in the study, is important. In the event of discrepancies between the pathologists' assessments during the preliminary phase, a consensus meeting was convened using a multiheaded microscope to collaboratively reach a consensus regarding the FS-STAS status. Figure 3, *A*, shows representative FS slide with hematoxylin–eosin staining.

### Establishment of Gold Standard (GS) STAS Status

To establish the GS STAS status, a consensus meeting was held with 3 pathologists. The pathologists were tasked with diagnosing STAS using permanent slides. Underscoring that these pathologists were blinded to the results of the FS evaluations and other clinicopathological information related to the patients is important. The FS-STAS and GS-STAS were evaluated separately and on different dates. Figure 3, *B*, shows a representative hematoxylin–eosin staining of a permanent slide.

### Statistical Analysis

The diagnostic performance of detecting STAS using FS slides was assessed by comparing FS-STAS and GS-STAS results. This comparison provided values for sensitivity, specificity, positive predictive value (PPV), negative predictive value (NPV), and accuracy.

## Results

### Patient Characteristics

Table 1 summarizes the characteristics of the 99 patients and 114 tumor lesions included in the study, including 84 primary lung cancers and 30 metastatic lung tumors. The median age of the patients was 73 years; the majority were male (60%) and former or current smokers (60%). Only 13% of the patients had a history of chronic obstructive lung disease. All patients underwent minimally invasive lung resection via video- or robot-assisted thoracic surgery.

**Table 1:** Demographics of the study cohort

Demographics	
Patient demographics	N = 99
Age, y	73 (66-77)
Sex	
Female	40 (40%)
Male	59 (60%)
Smoking	
Never	40 (40%)
Former/current	59 (60%)
COPD	
Absent	86 (87%)
Present	13 (13%)
Number of tumor lesions	
1	88 (89%)
2	7 (7%)
3	4 (4%)
Tumor characteristics	N = 114
Tumor location	
RUL	26 (23%)
RML	10 (9%)
RLL	28 (25%)
LUL	24 (21%)
LLL	26 (23%)
Tumor size, cm	1.7 (1.2-2.6)
Type of lung resection	
Wedge	29 (25%)
Segmentectomy	51 (45%)
Lobectomy	34 (30%)
Primary or metastasis	
Primary lung cancer	84 (74%)
Metastatic lung tumor	30 (26%)
Histology of primary lung cancer	
Adenocarcinoma	70 (61%)
Squamous cell carcinoma	9 (8%)
Large cell carcinoma	3 (3%)
Others	2 (2%)
Primary site of metastatic lung tumor	
Colorectum	15 (13%)
Uterus	6 (5%)
Ovary	3 (3%)
Kidney	2 (2%)
Others	4 (4%)
STAS	
Absent	71 (62%)
Present	43 (38%)

Data are shown as number (25-75 percentiles) or number (%). *COPD*, Chronic obstructive lung disease; *RUL*, right upper lobe; *RML*, right middle lobe; *RLL*, right lower lobe; *LUL*, left upper lobe; *LLL*, left lower lobe; *STAS*, spread through air spaces.

Of the 11 patients with multiple tumors, 7 had 2 tumors and 4 had 3. Of these patients, 5 had metastatic lung tumors and 6 had primary lung cancers. The treatment methods varied, with 6 patients undergoing a combination of segmentectomy and wedge resections, 2 undergoing a combination of lobectomy and wedge resections, and 3 undergoing multiple wedge resections.

In the 99 patients, 114 tumors were identified. Among them, 76 patients had 84 primary lung tumors, whereas the remaining 23 patients had 30 metastatic lung tumors. Adenocarcinoma was the most common histology in the primary lung cancers, accounting for 61% of all cases. Squamous cell carcinoma and large cell carcinoma were less frequent, at 8% and 3%, respectively. Most metastatic lung tumors originated from colorectal carcinoma, followed by ureteral (5%), ovarian (3%), and renal (2%) malignancies.

### Diagnostic Performance of FS Slides for Detecting STAS

Table 2 presents the diagnostic performance of the FS in detecting STAS. GS-STAS was positive in 43 of 114 total lesions (38%), 31 of 84 primary lung cancers (37%), and 12 of 30 metastatic lung tumors (40%). The diagnostic accuracy measures for STAS detection using FS slides for the 114 lesions, sensitivity, specificity, PPV, NPV, and overall accuracy, were 81%, 89%, 81%, 89%, and 86%, respectively. Regarding the 84 lesions of primary lung cancer, the sensitivity, specificity, PPV, NPV, and overall accuracy were 90%, 89%, 82%, 94%, and 89%, respectively. Finally, considering the 30 lesions of metastatic lung tumors, the sensitivity, specificity, PPV, NPV, and overall accuracy were 58%, 89%, 78%, 76%, and 77%, respectively. Regarding the STAS positivity using the FS analysis, of the 114 total lesions, 43 (37.7%) were found to have STAS. Among patients with primary lung cancer, STAS was observed in 34 of 84 cases (40.5%), whereas among metastatic tumors, it was found in 9 of 30 cases (30%). Table 3 lists the diagnostic performance metrics of each pathologist.

**Table 2:** Diagnostic performance for detecting STAS using FS slides

Cohort	GS-STAS+ (%)	FS-STAS+ (%)	STAS diagnostic performance using frozen sections				
			Sensitivity	Specificity	PPV	NPV	Accuracy
Total (n = 114)	43 (38%)	43 (38%)	81%	89%	81%	89%	86%
Primary LC (n = 84)	31 (37%)	34 (40%)	90%	89%	82%	94%	89%
Metastatic tumor (n = 30)	12 (40%)	9 (30%)	58%	89%	78%	76%	77%

GS-STAS+ is the designation for positive STAS status as determined by the GS analysis using permanent slides in a consensus meeting of 3 pathologists. FS-STAS + reflects STAS detection as determined by the cryo-embedding FS technique. *GS*, Gold standard; *STAS*, spread through air spaces; *FS*, frozen section; *PPV*, positive predictive value; *NPV*, negative predictive value; *LC*, lung cancer.

**Table 3:** Diagnostic performance of the individual examiners

Cohort	GS-STAS+ (%)	Examiners	FS-STAS+ (%)	STAS diagnostic performance using frozen sections				
				Sensitivity	Specificity	PPV	NPV	Accuracy
Total (n = 114)	43 (38%)	A	43 (38%)	67%	80%	67%	80%	75%
		B	39 (34%)	74%	90%	82%	85%	84%
		C	56 (49%)	81%	70%	63%	86%	75%
Primary LC (n = 84)	31 (37%)	A	32 (38%)	71%	81%	69%	83%	77%
		B	32 (38%)	90%	93%	88%	94%	92%
		C	42 (50%)	87%	72%	64%	91%	77%
Metastasis (n = 30)	12 (40%)	A	11 (37%)	58%	78%	64%	74%	70%
		B	9 (30%)	50%	83%	67%	71%	70%
		C	14 (47%)	67%	67%	57%	75%	67%

GS-STAS+ is the designation for positive STAS status as determined by the GS analysis using permanent slides in a consensus meeting of 3 pathologists. FS-STAS + reflects STAS detection as determined by the cryo-embedding FS technique. *GS*, Gold standard; *STAS*, spread through air spaces; *FS*, frozen section; *PPV*, positive predictive value; *NPV*, negative predictive value; *LC*, lung cancer.

## Discussion

In this prospective study, we evaluated the diagnostic performance of FS slides for STAS detection using the established cryo-embedding medium inflation technique. The novelty and strengths of this study are as follows. First, the cryo-embedding medium inflation technique significantly increased the sensitivity of STAS detection using FS slides. By expanding the lung parenchyma, this method may allow pathologists to improve differential tumor visualization from the surrounding tissues, leading to more accurate diagnoses of STAS. Second, creating cryo-embedded medium–injected specimens and subsequent FS slides does not require any specific techniques, equipment, or skills beyond those already implemented in institutions where FS analysis is performed. Consequently, our proposed method could be easily implemented in daily practice without requiring additional resources.

Our research has shown that the diagnostic performance for intraoperative detection of STAS could be enhanced using our method. In the total cohort, the sensitivity and specificity were 81% and 89%, respectively, whereas in lung cancer, they were 90% and 89%, respectively. Regarding previous benchmarks, low sensitivities ranging between 44% and 55% and specificities ranging between 80% and 91% have been reported.^7^^,^^8^ This highlights the need for improved techniques, particularly for increasing these low sensitivities. Our adapted cryo-embedding method demonstrated increased sensitivity and maintained high specificity. This suggests that our method could enhance intraoperative STAS detection and improve decision-making during surgery. However, several studies have evaluated novel technologies for predicting STAS preoperatively, such as radiomics or 3-dimensional convolutional neural networks, and found predictive values for preoperative STAS detection.^11^^,^^12^ Although these technologies may require specific equipment and skills, they could serve as potential alternatives for intraoperative histologic detection of STAS. Moreover, a combination of radiologic and intraoperative diagnostic methods could yield the most accurate results.

In addition, Table 3 presents notable intraobserver variability, which underscores the subjective nature of STAS detection on FS slides. Examiner A's sensitivity in primary lung cancer cases was 71%, compared with 90% and 87% of Examiner B and Examiner C, respectively. This variability may stem from individual differences in interpretation and highlight the need for establishing standardized criteria or training to minimize discrepancies in STAS detection.

Two recent large randomized trials revealed equivalent oncologic outcomes between lobectomy and sublobar resection.^2^^,^^3^ In patients with STAS-positive lung cancers, several studies have suggested that segmentectomy was associated with oncologic outcomes similar to those of lobectomy.^13^^,^^14^ Given that segmentectomy has superior functional and non-lung cancer–related outcomes than does lobectomy,^2^ it should not be deterred even in patients with STAS-positive lung cancers. However, a retrospective study on occult lymph node metastasis and its location suggested that STAS was strongly associated with a greater risk of intrapulmonary occult lymph node metastasis, with a significantly greater recurrence in patients who underwent wedge resection than in those who underwent anatomical lung resection.^15^ Therefore, if STAS is detected intraoperatively in patients intended to undergo wedge resection, anatomical lung resection approaches should be considered, including segmentectomy and lobectomy with hilar and mediastinal lymph node dissection, if functionally feasible.

Currently, the relationship between STAS status and selection of appropriate resection procedures in patients with metastatic tumors remains poorly understood. Our study did not assess the prognostic impact of STAS. However, previous findings, such as those from the study on aerogenous spread with floating cancer cell clusters in metastatic lesions,^16^ indicated that aerogenous spread was a significant factor in metastatic lung tumors and associated with local recurrence. This underscores the potential influence of STAS status on surgical decision-making, particularly in choosing between wedge and anatomical resections for metastatic tumors. Understanding the biological behavior of metastatic tumors in relation to STAS presence, including aspects such as aerogenous spread with floating cancer cell clusters, could provide valuable insights into optimizing surgical approaches and improving patient outcomes. This area requires further investigation, as it presents a promising avenue for future research to explore the implications of STAS in the context of metastatic lung cancer.

In this study, the sensitivity of the FS slide-based STAS detection in patients with metastatic lung tumors needs improvement, as our current sensitivity rate was only 58%. Despite this, our technique has clinical applicability beyond the sensitivity figures. It is crucial for physicians to have a technique that could be applied to both primary and metastatic lung tumors, specifically since indeterminate tumors are often encountered in real-world practice.

Although our study might provide insights into the diagnostic performance of STAS using the established cryo-embedding-medium inflation technique, highlighting several aspects of our study design that could affect its generalizability to routine clinical practice is important. First, the FS analysis was performed on a different day from that of surgery. This approach, despite being necessary to focus on the efficacy of the technique and avoid influencing actual treatment decisions, differs from the standard clinical practice, where FS analysis is typically performed intraoperatively. Second, the same pathologists were involved in both the FS and GS analyses. Although measures were taken to ensure independent evaluations and minimize bias, such as performing the FS analysis without previous knowledge of the GS results, this design may have still carried inherent limitations regarding potential bias. In addition, the pathologists were blinded to patient medical information to avoid bias, which may not have reflected the usual clinical scenario. These aspects, including the separation of FS analysis from the day of surgery, involvement of the same pathologists in both FS and GS analyses, and blinding of pathologists could potentially affect the generalizability of our findings. Future studies might consider incorporating a design that aligns more closely with standard intraoperative procedures and includes separate groups of pathologists for FS and GS analyses to validate our findings in a typical clinical setting.

In addition, this study has some limitations. First, the number of patients with metastatic lung tumors was small. Further research is warranted to determine the feasibility of applying the cryo-embedding medium inflation method for intraoperative detection of STAS to patients with metastatic lung tumors. Second, we did not record the time for the cryo-embedding process. Future studies are warranted to quantitatively assess the time efficiency of our method. Third, we did not evaluate the margin distance, which has been suggested to be associated with prognosis after resection in patients with STAS-positive tumors. Fourth, we were unable to compare the STAS status between FS control slides and other permanent slides within the same tumor owing to the pathologist blinding methods we used. As a result, our understanding of the variability of presence of STAS across different tumor sections could have been limited. To gain a deeper insight into the behavior of tumor cells in STAS-positive tumors, future research should focus on evaluating the consistency of STAS detection across tumor sections. Fifth, a notable limitation of our study could be the lack of historical data on STAS detection using traditional FS methods at our institution. Hence, our findings might not be directly compared with those of previous practices or literature. Our innovative approach may introduce unique biases that should be crucially considered when interpreting our results. Comparing our method with the traditional methods could provide a better understanding of the advantages and limitations of the cryo-embedding-medium inflation method in the future. Sixth, our study did not assess a formalized inter-rater reliability for the pathologists' evaluations of the FS specimens. This absence may have affected the consistency of our findings. Seventh, we used a 1:1 dilution of cryo-embedding medium and saline based on preliminary experiments, whereas a previous study has reported a 2:3 ratio.^10^ The different solution ratios might lead to varying outcomes, highlighting the need for standardization in future research.

## Conclusions

Incorporating the established cryo-embedding medium injection technique, we successfully expanded the lung parenchyma around tumor sites and enhanced STAS detection using FS slides, providing results similar to those of the GS STAS detection using permanent slides. The improved detection of STAS intraoperatively potentially can assist surgeons in making more informed intraoperative decisions, particularly regarding the appropriate extent of resection when segmentectomy or wedge resection is being contemplated. Selection of lobectomy, instead, when STAS is identified may lead to better overall oncologic outcomes.

## Conflict of Interest Statement

The authors reported no conflicts of interest.

The *Journal* policy requires editors and reviewers to disclose conflicts of interest and to decline handling or reviewing manuscripts for which they may have a conflict of interest. The editors and reviewers of this article have no conflicts of interest.
